# Improving sewage sludge compost process and quality by carbon sources addition

**DOI:** 10.1038/s41598-020-79443-3

**Published:** 2021-01-14

**Authors:** Liqiang Meng, Weiguang Li, Shumei Zhang, Xiancheng Zhang, Yi Zhao, Li Chen

**Affiliations:** 1 Institute of Microbiology, Heilongjiang Academy of Sciences, Harbin, 150010 China; 2 Institute of Advanced Technology, Heilongjiang Academy of Sciences, Harbin, 150020 China; 3 School of Environmental Engineering, Harbin Institute of Technology, Box no. 2602, 73 Huanghe Road, Harbin, 150090 Heilongjiang China; 4 State Key Laboratory of Urban Water Resource and Environment, Harbin Institute of Technology, Harbin, 150090 China

**Keywords:** Biochemistry, Biological techniques, Biotechnology

## Abstract

In present study, the effects of carbon sources on compost process and quality were evaluated in the lab-scale sewage sludge (SS) composting. The composting experiments were performed for 32 days in 5 L reactors. The results showed that carbon sources could change the nitrogen conversion and improve the compost quality. Especially, the readily degradable carbon source could promote organic matter degradation, improve nitrogen conversion process and accelerate compost maturation. The addition of glucose and sucrose could increase dissolved organic carbon, CO_2_ emission, dehydrogenase activity, nitrification and germination index during the SS composting. That's because glucose and sucrose could be quickly used by microbes as energy and carbon source substance to increase activity of microbes and ammonia assimilation. What's more, the NH_3_ emission was reduced by 26.9% and 32.1% in glucose and sucrose treatments, respectively. Therefore, the addition of readily degradable carbon source could reduce NH_3_ emission and improve compost maturity in the SS composting.

## Introduction

Sewage sludge (SS) is a by-product of wastewater treatment process, which contains a lot of N, P, K and other harmful components, such as heavy metals and pathogenic microorganisms^1^. With the expedited development of urbanization, the sewage treatment capacity is increasing year by year in China, which leads to large amounts of SS cannot be treated effectively in time^2,3^. China's urban SS is growing at a rate of 13% a year, with annual SS production expected to exceed 60 million tons by 2020, so the efficient and safe disposal of SS has become one of the main problems in China^4,5^. The urgency of SS treatment has been stressed in China's new environmental law and “*The Prevention and Control Action Plan of Water Pollution*”^6^.

Aerobic high-temperature composting is one of the effective ways to realize SS reduction, innocuity and reuse, furthermore, its products can be used as organic fertilizer and soil conditioner^7,8^. Admittedly, nitrogen was a key element in compost and could be recycled in agricultural application^9^. However, about 40%-80% nitrogen was lost through NH_3_ emission in the SS compost process, so much nitrogen loss must reduce compost quality and lead to air pollution^10,11^.

The low carbon–nitrogen ratio of SS is one of main reasons that lead to the severe NH_3_ emission in the composting process^12,13^. Therefore, in practical application, some substances rich in carbon sources are often added as bulking agent to improve the SS composting^14^. To increase the C/N ratio of substrates, lots of carbon-rich amendments, such as biochar, sawdust, spent mushroom substrate and cornstalks were added in composting^5,15–17^. However, the effects of different carbon sources amendment on NH_3_ emission was significantly different, even some amendment were not satisfying because of poor availability of the materials used^18^. That’s could be because the poor availability of amendment cause excessive nitrogen cannot be assimilated by microorganism and promote NH_3_ emission by microbial reactions^19,20^.

The previous research found that sucrose could improve the ammonia assimilation of microorganism and promote the transform of NH_4_^+^ into biological nitrogen^21,22^. Few studies of the effects of carbon sources types on NH_3_ emission in SS composting process have been performed. So, the main objectives of present research were to evaluate the influence of different carbon sources (glucose, sucrose, starch, and cellulose) on physical–chemical properties, organic matter degradation, dehydrogenase activity (DHA), NH_3_ emission, nitrification index and germination index in SS composting.

## Materials and methods

### Composting system

The dewatered SS were collected from a local sewage disposal plant in Harbin, China. Pumice was used as bulking agent, which was a light volcanic rock with no organic matter (OM). First 10 kg dewatered SS were mixed with 5 kg pumice, and then the mixture was divided into five parts. One of them was set as the control treatment without extra carbon source, and other four treatments were added 4% (carbon source/SS) sucrose, glucose, cellulose and starch. The composting material of each treatment was mixed evenly before the composting begins and put into respective reactors. The characteristics of raw materials were shown in Table 1. The reactors consisted of plastic cylinders with inner diameter 300 mm and height 600 mm respectively, and the other description were reported in previous literature^5^. In order to reduce the heat loss during composting, the reactors were put into a water tank whose temperature was set below 1∼3 °C of contol treatment^20^. The air was ventilated from the bottom of reactors by a air pump with 0.4 L/min aeration rate.

**Table 1:** The characteristics of the raw materials.

	pH	MC (%)	OM (%)
SS	7.34 ± 0.21	81.45 ± 1.12	56.23 ± 0.14
Pumice	6.83 ± 0.15	0.37 ± 0.21	0.56 ± 0.02
Glucose	7.04 ± 0.14	1.19 ± 0.10	99.34 ± 0.43
Sucrose	6.96 ± 0.54	0.34 ± 0.65	99.52 ± 0.09
Starch	6.83 ± 0.35	1.42 ± 0.15	98.87 ± 0.54
Cellulose	6.94 ± 0.28	0.54 ± 0.53	98.13 ± 0.45

The results are the mean ± standard deviation (n = 3).

*MC* moisture content, *OM* organic matter.

### Sampling protocol

The compost experiment was conducted for 32 days, and the samples were collected on Day 1, 3, 5, 7, 9, 13, 18, 23 and 32, respectively. The composting samples were collected from upper, middle and lower part of every reactor using the methods of quatering. The three 10 g samples were combined into one composite sample, after mixed thoroughly, the composite sample was divided into two parts on average. The first part was used to determine the moisture content through drying by an oven, and then the dried samples were used to determine organic matter (OM) content. The second part was stored at 4 °C for the measurement of dissolved organic carbon (DOC), DHA, NH_4_^+^, NO_3_^−^ and germination index, and all the samples were carried out in triplicate for variance analysis.

### Analysis methods

The OM contents were determined by measuring the loss of dry-solid mass after igniting at 550 °C for 5 h in a muffle furnace. The exhausted gas was obtain through a aluminium sampling bag everyday used the method of Maulini et al.^23^. The NH_3_ and CO_2_ were trapped by boric acid and natrium hydroxydatum solutions, respectively, then determined by titration^24^. The fresh sample was mixed with distilled water at 1:10 mass ratio (samples:distilled water) and oscillated in a shaker for 1 h. Then the mixture was centrifuged at 12,000 rpm for 5 min and the supernatant was filtered by 0.45 μm filter membrane to obtain the water extracts of sample^25^. The DOC content in the water extracts was measured by a Biotector TOC-B7000 TOC analyser (Hach, America). The DHA of sample was determined by the methods of Tiquia^26^. NH_4_^+^ and NO_3_^−^ were extracted in a 2 M KCl (sample at 1% mass ratio) and determined by colorimetry methods refer to the reports of Belyaeva and Haynes’^27^. The germination indexs (GI) were measured by pakchoi seeds and water extracts, twenty pakchoi seeds were distributed on the filter paper in a sterile dish (9 cm diameter), then 5 mL of the compost water extract was added to the filter paper and incubated at 20 ℃ for 3 days in dark. The computational method according to the author's previous method^5^. The data revealed in present paper were obtained from the average of three parallel samples and then were calculated by Microsoft Excel 2017 and the analyses of statistical were completed by SPSS 16.0.

## Results and discussion

### Organic matter and dehydrogenase activity

**Figure 1:**
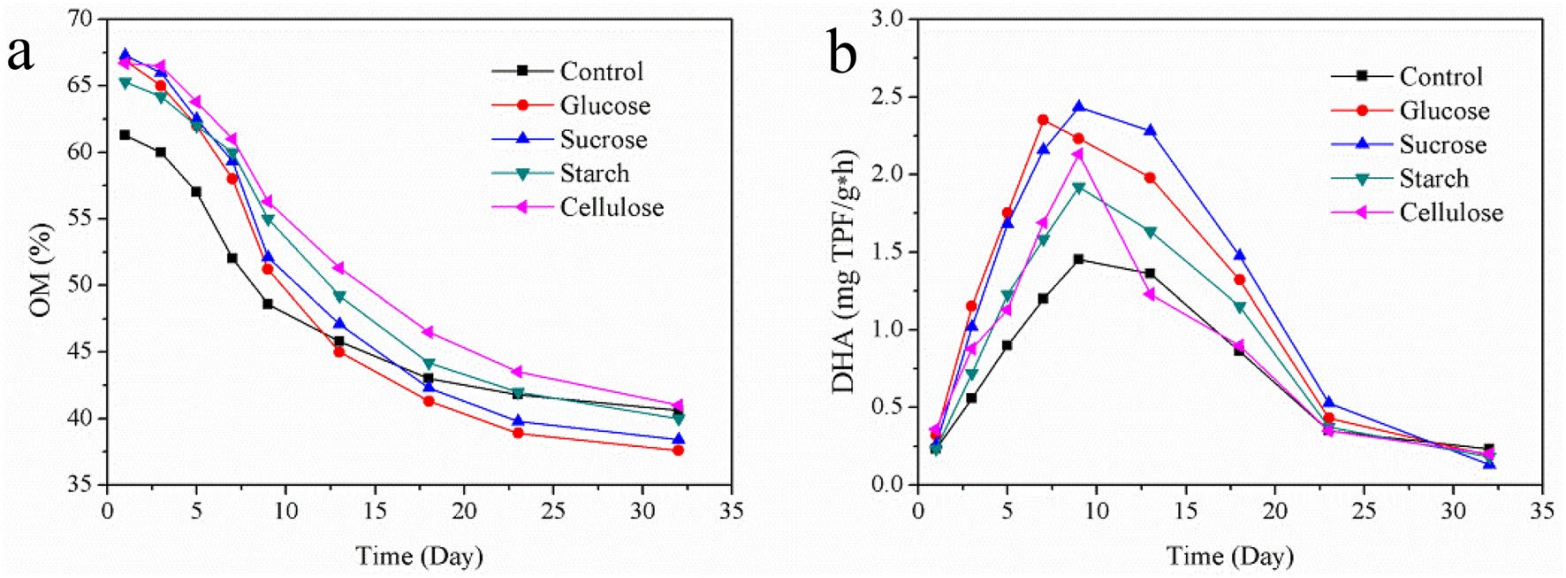
Evolutions of OM (**a**) and DHA (**b**) in five treatments.

The degradation rate of OM directly reflects the metabolism velocity of the microorganisms during the composting process^28^. The initial OM contents in carbon source treatment were higher than that in control treatment which was 61.3% (Fig. 1a). The OM contents in carbon source treatments were in the range of 65.3–67.3%, which showed the difference among them was not significant. The OM degraded rapidly of all treatments during the thermophilic phase of composting, and the OM contents dropped to 45.8% (control), 44.8% (glucose), 47.1% (sucrose), 49.2% (starch) and 51.3% (cellulose) respectively at the end of the thermophilic phase (Day 13). During mesophilic phase and thermophilic phase, the loss of OM contents in the control treatment and the cellulose treatment were 15.5% and 15.4%, respectively. However, the losses in glucose and the sucrose treatment were higher than the other treatments, were 21.9% and 20.2%, respectively. Moreover, the loss of starch treatment was slightly higher than that in control treatment was 16.1%. That’s because the glucose and sucrose belong to easily degradable carbon sources which were easy to be used by microorganisms, could increase the activity of composting microorganisms and promote the degradation of OM. Contrarily, owing to the complex macromolecular structure of cellulose that was not easy to be used by microorganisms, the addition of cellulose has the least impact on the degradation rate of OM^11^. At the end of composting, the lowest OM degradation rates also appeared in the glucose and the sucrose treatment, which were 37.6% and 38.4%, respectively. However, the differences between the other three treatments were not significant in the range of 40.1–41.2%. Obviously, the addition of glucose and sucrose would promote the metabolic activities of microorganisms and accelerate the degradation of OM in composting.

Dehydrogenase is a collective name for a series of metabolic reaction enzymes that catalyze the degradation of OM to produce ATP inside the microbial cells^29^. Therefore, DHA has been recognized as an important parameter that can react to the speed of biochemical reactions during composting^30^. The evolution of DHA is shown in Fig. 1b, the DHA of each treatment was extremely sensitive to temperature, what's more, they showed a positive correlation^31^. The DHA increased in the mesophilic phase and thermophilic phase in all treatments, and reached their peak values in the thermophilic phase, then the DHA gradually decreased in the cooling phase until the end of composting. The results showed that the addition of carbon source significantly increased the DHA, and the different raise between types of carbon sources were significant. The DHA of glucose treatment reached peak on Day 7 and the peak value of 2.35 mg TPF/g h, while the other four treatments reached the peak on Day 9. The peak value of sucrose treatment was 2.44 mg TPF/g h that was the highest, followed by the starch, cellulose and control treatment which were 1.92 mg TPF/g h, 2.13 mg TPF/g h, and 1.45 mg TPF/g h, respectively. Nikaeen et al.^30^ also reported that DHA had been increasing during the initial phase of composting, and then gradually decreased with decreasing temperature in the cooling phase. Previous reports had confirmed that DHA showed positive correlation with the culturable microorganisms in composting^5^, therefore, the addition of carbon sources could provide energy materials for the compost microorganisms, promote the microorganisms growth and improve DHA in compost. Similar phenomenon had been found by Zhang et al.^32^, who reported that the nutrients in the bulking agent could promote the metabolism of compost microorganisms and increase their biological activity and biomass. In present study, as readily degradable carbon source glucose and sucrose could be quickly used by microorganisms, so the DHA were stronger than those in other treatments, this results was consistent with previous reports^33,34^.

### Dissolved organic carbon and CO_2_ emission

**Figure 2:**
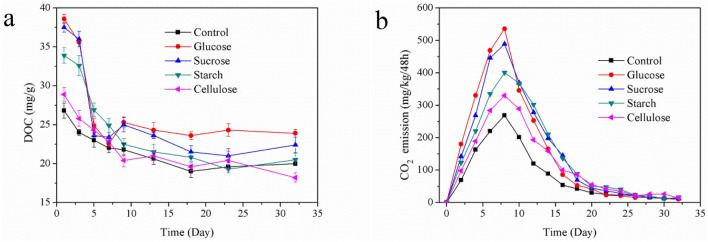
Evolutions of DOC (**a**) and CO_2_ emission (**b**) in five treatments.

DOC mainly includes small molecule, simple structure and water soluble carbon source materials, so it is easily used by microorganisms to participate in the biochemical reaction of composting^35^. The change of DOC in the composting could indirectly reflect the metabolism of compost microorganisms, and DOC had a certain relationship with the degradation of OM and the maturity of the compost product, so DOC could also be used to evaluate the stability of composting product^36^. The evolutions of DOC in five treatments are showed in Fig. 2a, the differences of initial DOC concentrations between five treatments were significant, which was closely related to the type of extra carbon source. The concentrations of DOC of the glucose and sucrose treatments were significantly higher than those of other treatments, because the glucose and sucrose were easily degradable carbon source, and their water solubility directly led to the increase in DOC concentration. However, the initial DOC concentration of starch treatment was lower than that of the glucose and sucrose treatments, and cellulose and control treatments had the lowest initial DOC concentrations, because the larger molecular structures in starch and cellulose could not be easily soluble in composting^20^. In the early stages (Day 1–7) of composting, the DOC concentration of all treatments decreased rapidly, especially in the glucose and sucrose treatments, which decreased from 38.6 mg/g and 37.5 mg/g to 22.6 mg/g and 23.4 mg/g, respectively. And the decreases were 16.0 mg/g and 14.1 mg/g, which were much higher than those of other treatments. In addition, the DOC concentrations of glucose and sucrose treatments experienced a slight increase on the Day 7–9 in thermophilic phase, this might be because the readily degradable carbon source promoted the degradation of the original OM of the SS composting and the release rate of DOC from OM degradation was higher than the degradation rate of it. The degradation of DOC in the cellulose treatment was similar to that of the control treatment, which was fast in the mesophilic phase and thermophilic phase of the compost. The DOC concentrations of all treatments remained stable during the cooling phase, and the final DOC concentration was in the range of 18.2–23.9 mg/g.

CO_2_ was generated during the degradation of OM by microorganisms in the composting, so the release rate of CO_2_ could reflect the activity of microorganisms and the degradation rate of OM^37^. The evolutions of the CO_2_ emission in the five treatments are shown in Fig. 2b. The change trends of CO_2_ emission in all treatments were similar, but the amounts of CO_2_ emission were significantly different. All the amounts of CO_2_ emission increased rapidly in mesophilic and thermophilic phase, and reached their respective peaks on the Day 8 of composting. Thereinto, the biggest CO_2_ emission amount was 536.3 mg/kg/48 h in glucose treatment, followed by 489.6 mg/kg/48 h in sucrose treatment, 401.2 mg/kg/48 h in starch treatment, 330.6 mg/kg/48 h in cellulose treatment and 269.5 mg/kg/48 h in control treatment. After the peaks, the amount of CO_2_ emission in each treatment gradually decreased until the end of composting, and only a small amount of CO_2_ was volatilized during Day 20–32 of composting. Obviously, the addition of extra carbon source promotes the CO_2_ emission during the composting process. What’s more, as easily degradable carbon source, glucose and sucrose were easier to be used by microorganisms, whose promotions for CO_2_ emission were stronger than those in other treatments. The accumulative amounts of CO_2_ emission were 2557.2 mg/kg and 2576.6 mg/kg in glucose and sucrose treatments, which were 86.9% and 88.3% higher than that in the control treatment at 1368.2 mg/kg, respectively. The accumulative amounts of CO_2_ emission in starch and cellulose treatments were 2391.6 mg/kg and 1947.2 mg/kg, respectively, which were lower than those in the glucose and sucrose treatments and higher 74.8% and 42.3% than the control treatment. Because the complex macromolecular structure of cellulose was not easily used by microorganisms, the promotion of CO_2_ emission was negligible. Moreover, starch was easier to be used by microorganisms than cellulose, so the CO_2_ emission in starch treatment was higher than that in cellulose treatment.

### NH_4_^+^, NH_3_ emission, NO_3_^−^, Nitrification index

**Figure 3:**
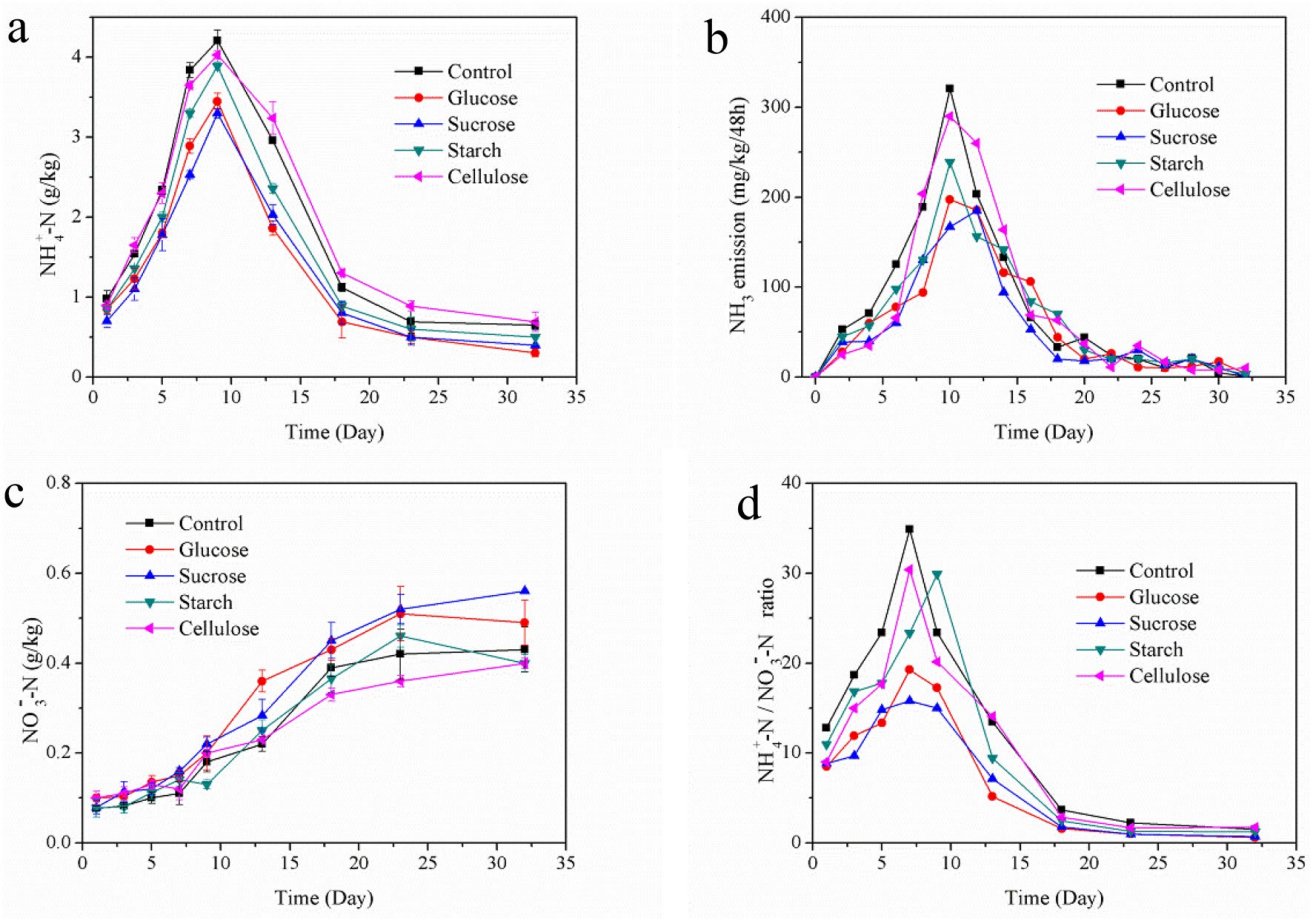
Evolution of NH_4_^+^-N (**a**), NH_3_ emission (**b**), NO_3_^–^N (**c**) and NH_4_^+^-N/NO_3_^–^N (**d**) in five treatments.

NH_4_^+^ would be produced through the ammonification of microorganisms with OM degradation in the SS composting process^27^. The evolutions of NH_4_^+^ are shown in Fig. 3a, the NH_4_^+^ concentrations of all treatments increased rapidly during the mesophilic phase and reached their respective peaks on the Day 9 of thermophilic phase. In besides, the change trend of each treatment was similar, because the increase in NH_4_^+^ concentration was mainly caused by the rapid degradation of nitrogen-containing organic compounds^38^. The NH_4_^+^ concentrations of control treatment were significantly higher than those of the carbon source treatments, and it’s peak value was 4.21 g/kg, which were 1.22, 1.28, 1.08 and 1.04 times of that in glucose, sucrose, starch, and cellulose treatments, respectively. In the mesophilic phase, with the temperature increased the microorganisms multiplied rapidly, the DHA also increased rapidly (introduced in “Organic matter and dehydrogenase activity”) and the ammonification of microorganisms played a major role in nitrogen conversion process. The peak concentrations of NH_4_^+^ of the glucose and sucrose treatments were much lower than that of the control treatment, because the microorganisms could more easily use the degradable carbon sources and promote the ammonia assimilation, that transformed the NH_4_^+^-N into biological nitrogen. On the contrary, the contents of DOC in the cellulose and control treatments were lower than the other treatments (introduced in “Dissolved organic carbon and CO_2_ emission”), so a large amount of nitrogen were converted into NH_4_^+^ by ammonification.

After the peak values, the NH_4_^+^ concentrations in all treatments decreased rapidly, because high temperature and the gradually rising pH value promoted the conversion of NH_4_^+^ to NH_3_ and released into the atmosphere eventually^24^. Only a small NH_4_^+^ could be detected at the end of composting, and the NH_4_^+^ concentration in control and the cellulose treatments were slightly higher than those in other treatments, which were 0.65 g/kg and 0.69 g/kg, respectively. Ros et al.^39^ also reported that the NH_4_^+^ concentration gradually increased during the first 3–6 weeks of composting, and then gradually decreased due to NH_3_ emission until the end of composting.

NH_3_ emission is one of the main reasons for the nitrogen loss, and the too low carbon–nitrogen ratio of the sewage sludge is an important factor that causes a large amount of NH_3_ emission during SS composting process^40^. Adding extra carbon source could effectively control nitrogen loss in SS composting^22^, however, the effects of different carbon sources on NH_3_ emission were different.

The NH_3_ emission rate increased rapidly with the temperature rising in the early stage, then reached their respective peaks in thermophilic phase. The peak value of NH_3_ emission of control treatment was 320.7 mg/kg/48 h, followed by 290.6 mg/kg/48 h in cellulose treatment, 239.5 mg/kg/48 h in starch treatment, 197.5 mg/kg/48 h in glucose treatment and 185.8 mg/kg/48 h in sucrose treatment (Fig. 3b). The emission rate of NH_3_ had a positive correlation with the concentration of NH_4_^+^ in the composting. Therefore, the NH_3_ emission amount of each treatment increased sharply when the concentrations of NH_4_^+^-N were high in the thermophilic phase, and the NH_3_ emission was also gradually flattening when the concentration of NH_4_^+^ decreased in the cooling phase. The addition of carbon source changed the metabolic pathway of nitrogen, concretely, it could affect the microbial ammonification which led directly to NH_3_ emission during the composting process. For example, the peak of the sucrose treatment appeared on Day 12, and the peaks of the other 4 treatments appeared on Day 10. Moreover, the addition of extra carbon source significantly inhibited the NH_3_ emission, especially in the readily degradable carbon source treatments, the NH_3_ emission peaks were reduced by 38.4% and 42.3% in glucose and sucrose treatments compared with the control treatment, respectively.

In the whole composting process, the cumulative NH_3_ emission of sucrose treatment was 900.3 mg/kg, which was the least of five treatments, followed by 968.8 mg/kg in glucose treatment, 1168.5 mg/kg in starch treatment, 1278.3 mg/kg in cellulose treatment and 1325.9 mg/kg in control treatment. In addition, the cumulative NH_3_ emission was decreased by 32.1%, 26.9%, 11.8% and 3.6% compared with the control treatment in sucrose, glucose, starch and cellulose treatments, respectively. That’s because glucose and sucrose were easily used by microorganisms, and could participate in the biochemical reaction of composting faster. Moreover, the readily degradable carbon source could increase the DOC concentration (introduced in “Dissolved organic carbon and CO_2_ emission”) and improve the biodegradability of the carbon source, so more NH_4_^+^ was converted to bio-nitrogen fixed in the compost.

The nitrification in the composting process could be evaluated by detecting the NO_3_^−^ concentration. As shown in Fig. 3c, the NO_3_^−^ concentrations of all the treatments were lower than the NH_4_^+^ concentrations throughout the composting process, especially during mesophilic phase and thermophilic phase. The NO_3_^−^ concentration was closely related to the activity of nitrifying microorganisms, but the activity of nitrifying microorganisms was inhibited by high temperature and high NH_4_^+^ concentration, that's why the NO_3_^−^ concentration were so low during the thermophilic phase. During the first 7 days of composting, the NO_3_^−^ concentration of each treatment was at a low level (0.07–0.16 g/kg). The NO_3_^−^ concentrations in carbon source treatment was slightly higher than that in control treatment, then difference among the carbon source treatments was not significant. During the cooling phase, the NO_3_^−^ concentrations of all treatments began to increase as the temperature decreased. The NO_3_^−^ concentrations of glucose and sucrose treatment was significantly higher than that of control, while the differences among starch, cellulose and control treatment were not significant. The NO_3_^−^ concentrations of all treatments reached a stable state at end of composting, and the highest NO_3_^−^ concentration was 0.56 g/kg in sucrose treatment, followed by 0.43 g/kg in glucose treatment, 0.31 g/kg in starch treatment, 0.24 g/kg in cellulose treatment and 0.25 g/kg in control treatment. With the decrease of temperature and the NH_3_ emission in the cooling phase, the increase of nitrification in all the treatments caused NO_3_^−^ concentration raised, and the difference between glucose and sucrose treatments was not significant. Their change trends were similar, could be because the glucose and sucrose as easily degradable carbon sources were exhausted during mesophilic phase and thermophilic phase.

The nitrification index was defined as the ratio of NH_4_^+^ to NO_3_^−^, which had been widely used in the evaluation of compost maturity^41^. As shown in Fig. 3d, the nitrification index of each treatment reached its peak in thermophilic phase, that’s because the OM rapidly degraded during this period and the NH_4_^+^ concentration remained at a high level. After the thermophilic phase, the nitrification index decreased rapidly as the temperature decreased, because the degradation rate of OM became slower, and the compost gradually completed maturation. Moreover, all the treatments reached maturity on Day 23 of the composting. According to Das et al.’s reports, the nitrification index of less than 0.5 indicated that the compost had reached "completely mature" state at the end of composting, the nitration index between 0.5 and 3.0 indicated that the compost had reached a "mature" state, and the nitrification index greater than 3.0 indicated that the compost had not reached a mature. In present study, all carbon source treatments reached maturity on Day 18 of composting. And the minimum nitrification index was 1.60 in glucose treatment, followed by 1.78 in sucrose treatment, 2.43 in starch treatment and 2.85 in cellulose treatment, while the control treatment was still in immature state with a nitration index of 3.69.

### Germination index

**Figure 4:**
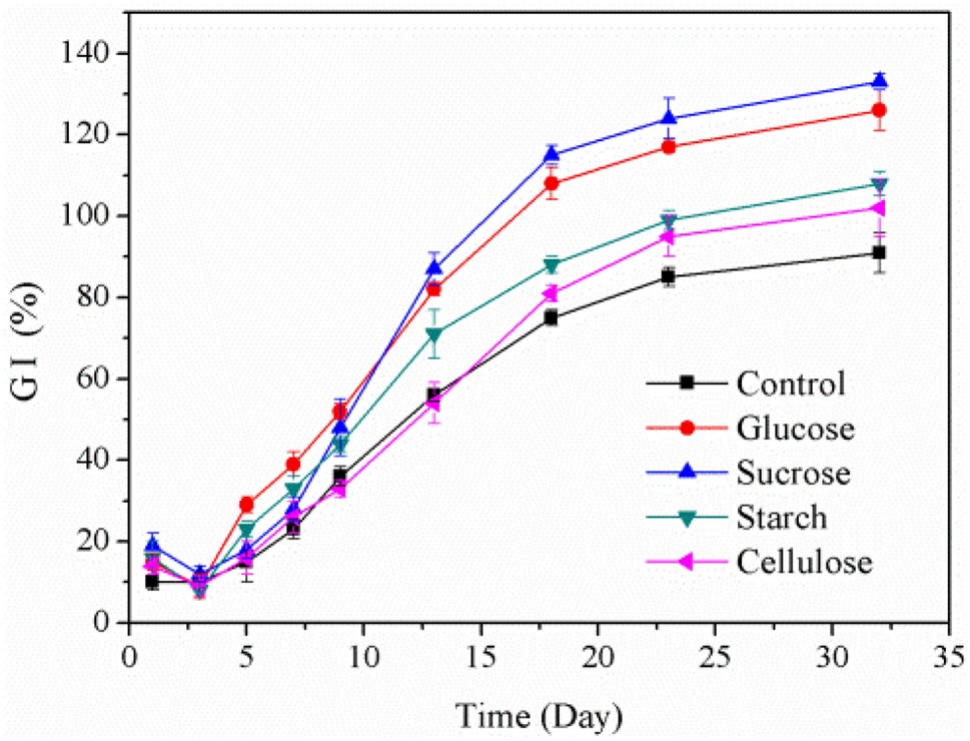
Evolution of GI in five treatments.

Germination index (GI) is a biological activity index commonly used to evaluate compost maturity, and it can intuitively reflect the phytotoxicity change of compost. As shown in Fig. 4, all the GI decreased during first two days of composting, which was due to the production of some NH_4_^+^ and low-molecular-weight short-chain volatile fatty acids in the early stage of composting. Similar phenomenon had been found by Guo et al.^42^, who reported that the seed GI was at a low level in early stage when they studied the co-composting of corn straw and pig manure. It’s reported that when the GI of the compost material exceeded 80%, it indicated that the compost product had no phytotoxicity and reached the maturity state^43^. The GI of all treatments increased rapidly after Day 3 of composting, which might be due to toxic substances degradation and NH_3_ emission. Importantly, the GI of the carbon source treatment was higher than that of control, then GI of the carbon source treatments reached over 80% on Day 18 of composting, concretely, the highest GI was 115.2% in sucrose treatment, followed by 108.9% in glucose treatment, 88.3% in starch treatment, 81.6% in cellulose treatment, and only 74.7% in control treatment. The GI of all treatments exceeded 80% on Day 23 of composting, indicating all the treatments reached no phytotoxicity and mature state. Obviously, the addition of extra carbon sources, especially easily degradable carbon sources, could promote the compost maturation, and the compost could dephytotoxicize earlier.

## Conclusions

The addition of extra carbon sources could improve the nitrogen conversion and compost quality. Especially, glucose and sucrose, as readily degradable carbon source promoted OM degradation and the maturation of composting, increased DOC contents, CO_2_ emission, DHA, nitrification index and GI in the SS compost. Above all, the addition of sucrose and glucose reduced the NH_3_ emission by 32.1% and 26.9%, respectively. Nevertheless, it’s recommended that suitable alternatives for glucose and sucrose, such as beet pulp and molasses wastes containing readily degradable carbon sources should be researched to decrease production costs in future research.
